# qtl.outbred: Interfacing outbred line cross data with the R/qtl mapping software

**DOI:** 10.1186/1756-0500-4-154

**Published:** 2011-05-26

**Authors:** Ronald M Nelson, Xia Shen, Örjan Carlborg

**Affiliations:** 1 Department of Breeding and Genetics, Swedish University of Agricultural Sciences, Box 7023, SE-75007 Uppsala, Sweden; 2 Department of Cell and Molecular Biology, Uppsala University, BMC Box 598, SE-75124 Uppsala, Sweden

## Abstract

**Background:**

**qtl.outbred **is an extendible interface in the statistical environment, R, for combining quantitative trait loci (QTL) mapping tools. It is built as an umbrella package that enables outbred genotype probabilities to be calculated and/or imported into the software package R/**qtl**.

**Findings:**

Using **qtl.outbred**, the genotype probabilities from outbred line cross data can be calculated by interfacing with a new and efficient algorithm developed for analyzing arbitrarily large datasets (included in the package) or imported from other sources such as the web-based tool, GridQTL.

**Conclusion:**

**qtl.outbred **will improve the speed for calculating probabilities and the ability to analyse large future datasets. This package enables the user to analyse outbred line cross data accurately, but with similar effort than inbred line cross data.

## Background

QTL mapping is one of the first steps towards understanding the genetic basis of complex traits. With the rapid advances in genotyping technologies it is common to obtain dense genotype data for many individuals. While there are a number of software packages available for QTL mapping and analysis, none can currently handle large datasets for outbred line crosses. Here, we introduce a software package, **qtl.outbred**, which provides an interface between outbred line cross data and the popular QTL mapping and analysis tool, R/**qtl **[1].

**qtl.outbred **enables the user to convert and import genotype probabilities from outbred line crosses to R/**qtl **in the freely available R environment [2]. After importing the genotype probabilities, the functions in R/**qtl **downstream of its native calc.genprob function can be used on the imported data. **qtl.outbred **also provides a function for calculating genotype probabilities from outbred line cross data for large datasets using a newly developed and computationally highly efficient algorithm [3]. Alternatively, output from other software, e.g. GridQTL [4], can be ported directly for analysis in R/**qtl**.

The purpose of this interface is to make the established mapping tools in R/**qtl**, originally developed for inbred line cross data, available to the wider scientific community. The package focuses on outbred line cross datasets, which are often found in research on agricultural plants and animals, selection lines in experimental species including mice, as well as for a number of animal models of human diseases.

## Implementation

The functions in **qtl.outbred **are summarized in Table 1. The main task of **qtl.outbred **is to import genotype probabilities, calculated for outbred cross data, into the R-environment and converting them into an object of the class *cross *in R/**qtl**. This data object can then directly be used in the R/**qtl **package [1] for further QTL mapping analyses. R/**qtl **is well established and provides a comprehensive set of tools for QTL analysis which are applicable to all type of line cross data (once the genotype probabilities are calculated and imported), but today this software is limited to inbred line cross data. Importing outbred line cross data through **qtl.outbred **provides access to these tools to users with outbred line cross data.

**Table 1:** Functions in the package **qtl.outbred**.

Function	Description
calc.prob	Calculating genotype probabilities using the triM algorithm
impo.prob	Importing calculated genotype probabilities to R/qtl

Calculation of genotype probabilities from outbred line cross data is not trivial and **qtl.outbred **provides support for obtaining these values. We recommend using the build-in function in **qtl.outbred**, which is much faster and more accurate than the current methods [5]. This method uses a new algorithm (triM) that calculates genotype probabilities from marker and pedigree data from F2 and back-cross populations, using a hidden Markov model [3].

Other features in **qtl.outbred **include the option to directly import genotype probabilities generated from the widely used GridQTL software [4]. However, the simple input format used in **qtl.outbred**, should allow the user to create input files from any other files with genotype probability data.

## Results

**Figure 1:**
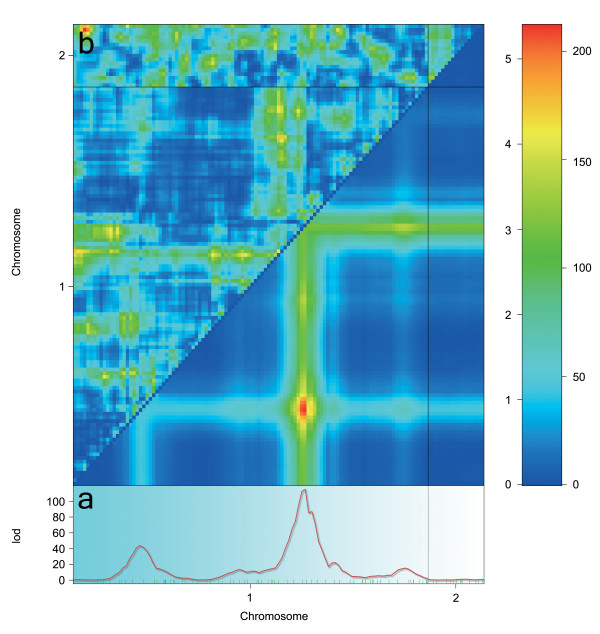
**The graph was obtained by using outbred line cross data (domesticated and wild chicken intercross genotypic data with simulated phenotype), calculating genotype probabilities with the triM algorithm from the qtl.outbred interface and importing it directly to R/qtl where the genome scans were performed**. LOD scores for Haley-Knott regression [6] for (a) single-QTL genome scan and (b) two-QTL genome scan are reported. LOD scores are indicated on the colour scale where, numbers to the left correspond to the upper triangle indicating two-locus epistasis and values to the right correspond to the lower triangle indicating the significance for a test of two versus one QTL.

**qtl.outbred **has been extensively tested. Firstly, we established that the triM algorithm produce exactly the same genotype probabilities as R/**qtl **when inbred line cross data are used (i.e. line crosses of inbred mouse strains). Secondly, we used genotypic data from an outbred line cross between domesticated and wild chickens with a simulated phenotype. Genotype probabilities were calculated with the triM algorithm using **qtl.outbred **to interface it with R/**qtl**. The single- and two-QTL genome scan for this dataset is illustrated in Figure 1. The identified peaks correspond to where the QTL were simulated. Lastly, we calculated QTL genotype probabilities for the simulated chicken intercross using GridQTL. These genotype probabilities were imported in R/**qtl**, using the **qtl.outbred **interface, and the conducted QTL scan gave similar results to those reported in Figure 1.

## Conclusion

The purpose of **qtl.outbred **is to: 1. serve as an interface between the established software packages GridQTL and R/**qtl**; 2. provide an alternative to calculate QTL genotype probabilities in outbred crosses faster and more accurate than current software via the triM algorithm, and interface the results with R/**qtl**; 3. enable genotype probabilities calculated via any other method for outbred line cross to be imported into R/**qtl**; 4. provide these functions in a user friendly environment. This package is designed to fill the need for a fast and efficient QTL mapping environment for large datasets for outbred line crosses.

## Availability and Requirements

Project name: qtl.outbred

Project home page: https://r-forge.r-project.org/R/?group_id=844

Operating systems: Windows, Unix-like (Linux, Mac OSX)

Programming language: R, C/C++, Perl

Other requirements: R, Perl

License: GNU GPL

Any restrictions to use by non-academics: None.

## Competing interests

The authors declare that they have no competing interests.

## Authors' contributions

RMN, XS and C developed the concept. RMN and XS wrote the software and performed data analysis. RMN and ÖC wrote the manuscript. All authors read and approved the final manuscript.
